# Estimation of future suicide risk in psychiatric inpatiens with 6-item questionnaire

**DOI:** 10.1192/j.eurpsy.2022.337

**Published:** 2022-09-01

**Authors:** Z. Bélteczki, J. Újvári, Z. Rihmer

**Affiliations:** 1 Sántha Kálmán Psychiatric Hospital, I. Department, Nagykálló, Hungary; 2 Sántha Kálmán Psychiatric Hospital, I. Department, Nagykálló, Hungary; 3 National Institute of Menthal Health, Neurology and Neurosurgery, Nyírő Gyula Hospital, Budapest, Hungary

**Keywords:** suicide, questionnaire, prediction, risk

## Abstract

**Introduction:**

Estimation of suicide risk is difficult task, and the clinical utility of different suicide risk scales is far from ideal.

**Objectives:**

Previously we developed a 6-item clinician rated (yes/no) questionnaire (score range:0-28) that is able to detect current and past suicide risk with high sensitivity and specificity among acutely admitted psychiatric inpatients (Rihmer et al, 2017).

**Methods:**

The 151 (75 suicidal and 76 non-suicidial) psychiatric inpatients, admitted between 1 November 2016 and 31 March 2017 were followed till 31 August 2021. Cases of completed suicides and suicide attempters receiving medical attention were recorded.

**Results:**

During the 53-month follow-up 3 patients (2%) completed suicide (a 46 year old male with bipolar II disorder, a 57 year old female with schizoaffective disorder, a 55 year old male with schizoaffective disorder). Both of them were at baseline among the 75 suicidal inpatients and belonged to the group of “Marked suicide risk” (range:16-28 points) and scored 28,26 and 25 points, respectively. Suicide attempts have been made by 6 patients, all of them belonged to initially “Marked suicide risk” group (one initially non-suicidal, 16 points; 5 initially suicidal 22,26,26,26 and 28 points, respectively). 141 from the 151 patients received regular personal and/or on-line psychiatric care (including patients who died by suicide).

**Conclusions:**

Despite the small number of suicidal cases, our results suggest that this short, simple questionnaire might be helpful not only in detecting current and past suicidality, but also predicting future risk among discharged psyciatric inpatients.

**Disclosure:**

No significant relationships.

